# Married or Partnered Adults’ Self-Perceptions of Aging in Later Life: The Context of Gender

**DOI:** 10.1093/geroni/igaa057.2112

**Published:** 2020-12-16

**Authors:** Yijung Kim, Kyungmin Kim, Shevaun Neupert, Kathrin Boerner

**Affiliations:** 1 University of Massachusetts Boston, Boston, Massachusetts, United States; 2 North Carolina State University, Raleigh, North Carolina, United States

## Abstract

Marriage or other types of partnerships are consequential for health in later life, but its association to self-perceptions of aging remains a relatively unexplored area of research. This study used three waves of panel data from the Health and Retirement Study (N = 4,315) to examine how changes in the health status and relationship quality over time contribute to self-perceptions of aging for married/partnered men and women. Multilevel models showed that women demonstrated more positive self-perceptions of aging than men, but there was no gender difference in how self-perceptions of aging became more negative over time. The findings on the main and moderating effects of health and relationship quality give evidence that changes across time, as well as average differences in individual characteristics, may affect self-perceptions of married/partnered men and women differently. The context in which gender shapes key aspects of life contributes to self-perceptions of aging in later life.

